# Simultaneous Silencing of Two Arginine Decarboxylase Genes Alters Development in Arabidopsis

**DOI:** 10.3389/fpls.2016.00300

**Published:** 2016-03-14

**Authors:** Diana Sánchez-Rangel, Ana I. Chávez-Martínez, Aída A. Rodríguez-Hernández, Israel Maruri-López, Kaoru Urano, Kazuo Shinozaki, Juan F. Jiménez-Bremont

**Affiliations:** ^1^Laboratorio de Estudios Moleculares de Respuesta a Estrés en Plantas, División de Biología Molecular, Instituto Potosino de Investigación Científica y Tecnológica ACSan Luis Potosí, Mexico; ^2^Gene Discovery Research Group, RIKEN Center for Sustainable Resource ScienceTsukuba, Japan

**Keywords:** artificial microRNA, *Arabidopsis thaliana*, arginine decarboxylase, polyamines, development, seed

## Abstract

Polyamines (PAs) are small aliphatic polycations that are found ubiquitously in all organisms. In plants, PAs are involved in diverse biological processes such as growth, development, and stress responses. In *Arabidopsis thaliana*, the arginine decarboxylase enzymes (ADC1 and 2) catalyze the first step of PA biosynthesis. For a better understanding of PA biological functions, mutants in PA biosynthesis have been generated; however, the double *adc1*/*adc2* mutant is not viable in *A. thaliana*. In this study, we generated non-lethal *A. thaliana* lines through an artificial microRNA that simultaneously silenced the two *ADC* genes (amiR:*ADC*). The generated transgenic lines (amiR:*ADC*-L1 and -L2) showed reduced *AtADC1* and *AtADC2* transcript levels. For further analyses the amiR:*ADC*-L2 line was selected. We found that the amiR:*ADC*-L2 line showed a significant decrease of their PA levels. The co-silencing revealed a stunted growth in *A. thaliana* seedlings, plantlets and delay in its flowering rate; these phenotypes were reverted with PA treatment. In addition, amiR:*ADC*-L2 plants displayed two seed phenotypes, such as yellow and brownish seeds. The yellow mutant seeds were smaller than *adc1, adc2* mutants and wild type seeds; however, the brownish were the smallest seeds with arrested embryos at the torpedo stage. These data reinforce the importance of PA homeostasis in the plant development processes.

## Introduction

In plants, the growth and development processes are tightly regulated through a great variety of molecules with specific functions in a dynamic scenario. Within these molecules, the polyamines (PAs) are recognized as critical regulators in both important processes. Additionally, the PAs also modulate stress responses in plants (Wimalasekera et al., 2011; Gupta et al., 2013; Minocha et al., 2014).

The PAs are small, positively charges, organic molecules; and the mainly four ubiquitous PAs in plants are: diamine putrescine (Put), triamine spermidine (Spd), tetraamines spermine (Spm), and thermospermine (tSpm). Put is synthesized either directly from ornithine by ornithine decarboxylase (ODC) or from arginine by three sequential reactions catalyzed by arginine decarboxylase (ADC), agmatine iminohydrolase (AIH), and N-carbamoylputrescine amidohydrolase (NCPAH). Particularly, in *Arabidopsis thaliana*, the gene encoding ODC is absent (Hanfrey et al., 2001; Jiménez-Bremont et al., 2004) so it seems that the ADC pathway is the mainly route for PA biosynthesis in this plant. Put, is converted into Spd and Spm by the consecutive action of the spermidine synthase (SPDS) and spermine synthase (SPMS), respectively. Both reactions, catalyze the transfer of an aminopropyl group provided by decarboxylated S-adenosylmethionine (dcSAM), which is formed by S-adenosylmethionine decarboxylase (SAMDC) using S-adenosylmethionine (SAM) as a substrate (Kusano et al., 2008). An additional aminopropyl transferase named ACAULIS5 (ACL5), which is present only in plants and some prokaryotic organisms can synthesize the tetraamine tSpm (Minguet et al., 2008).

To elucidate the function of the PAs in diverse biological processes their levels have been manipulated; this can be achieved by biochemical or genetic strategies. In the first one, the use of inhibitors to critical enzymes in PA biosynthesis or application of exogenous PAs has been widely used. However, the use of inhibitors has certain limitations because a lot of them are non-specific being responsible for side effect responses. The second strategy has been generated by both loss-function mutants and gain-function transgenic plants of key enzymes of PA biosynthesis and other enzymes of PA metabolism (Mattoo et al., 2010; Hussain et al., 2011; Tiburcio et al., 2014).

*A. thaliana* has two *ADC* genes: *AtADC1* (AT2G16500) and *AtADC2* (AT4G34710), both encode functional enzymes that catalyze the first rate limiting step in the PA biosynthesis so its manipulation is critical to obtain knowledge of PAs function during plant development. In the latest 90′s, Soyka and Heyer (1999), reported that *A. thaliana* loss-function mutant of *AtADC2* exhibits no obvious phenotype under normal growth conditions but it was impaired to increase the ADC activity by osmotic stress in comparison to the wild type. Moreover, Urano et al. (2004) reported another *A. thaliana* mutant plant of *AtADC2* gene, which was more sensitive to salt stress than wild type plants. Finally, Urano et al. (2005) performed a double mutant of *AtADC1* and *AtADC2*, and reported that both genes are essential for seed development since the double mutant dies during the embryo stage. In all the previous report, it has been inferred a certain degree of functional redundancy between the two isoforms because mostly no obvious phenotype during normal conditions of the single mutants was observed, and since the PA levels did not suffer a drastic change (Soyka and Heyer, 1999; Urano et al., 2004). Nevertheless, the scenario is more complicated because it seems that *A. thaliana ADC* genes are differentially regulated, since *AtADC1* is constitutively expressed, and *AtADC2* is responsive to abiotic stresses, such as salinity, drought, wounding, ABA, MeJA, K^+^ deficiency, and bacterial pathogens (Takahashi and Tong, 2015).

In this study, we performed a simultaneous silencing of the two *ADC* paralogous genes in *A. thaliana* targeting a consensus region between both genes by an artificial microRNA (amiR:*ADC*). We explored whether the silencing led to a decrease in PA levels and the impact in *A. thaliana* development. Our observations suggest that the silencing strategy was successful due to significant lower expression levels of *AtADC1* and *AtADC2*, and consequently a drastic decrease in PA levels. Furthermore, we compared the amiR:*ADC* line with parental (Ws) and single *adc1* and *adc2* mutants. Excitingly, amiR:*ADC* line showed several development defects, such as stunted growth, including a delay in flowering rate, and morphology alteration of seed coat and viability. Our observations suggest that *ADC* paralogs in *A. thaliana* play a crucial role in plant growth and development during life span.

## Materials and methods

### Plant material and growth conditions

Seeds of *A. thaliana* ecotype Wassilewskija (Ws), and the T-DNA insertional mutants for *AtADC1* (*adc1*) and *AtADC2* (*adc2*) were used (Urano et al., 2005). First, the seeds were surface-sterilized for 7 min with 20% (v/v) chlorine solution and then washed four times with sterile distilled water. Next, the seeds were germinated and grown on Murashige and Skoog (MS) 0.5X plates, pH 5.7, containing 0.5% (w/v) sucrose, and 1.2% (w/v) agar (Murashige and Skoog, 1962). Seeds were stratified for 2 days at 4°C in the dark, and then the plates were incubated at 22 ± 2°C in a growth chamber with a 16 h light (120 μmol m^−2^ s^−1^) and 8 h darkness.

### AmiRNA design, vector construction, and plant transformation

The full-length nucleotide sequences of *AtADC1* and *AtADC2* transcripts were used to generate the artificial microRNA in the Web MicroRNA Designer (http://wmd3.weigelworld.org). The amiR:*ADC* is targeted of a consensus region of 21 nucleotides between the *AtADC* genes (*AtADC1*:1418–1438 and *AtADC2*:1535–1555 nt, respectively). The amiR:*ADC* was introduced into the *A. thaliana* miR319a precursor sequence by an overlapping PCR, as previously described by Schwab et al. (2006) and Ossowski et al. (2008). For this purpose, the pRS300 vector was used as a template and specific oligonucleotides were employed (Supplemental Table 1). The amiR:*ADC* precursor was cloned into pCR8/GW/TOPO entry vector (Invitrogen, Carlsbad, CA, USA) and sub-cloned into the pMDC32 binary vector (Curtis et al., 1994). The cloning was performed by site-specific recombination using Gateway LR Clonase II Enzyme Mix (Invitrogen). Finally, pMDC32-amiR:*ADC* construction was transformed into *Agrobacterium tumefaciens* GV2260 strain to generate *A. thaliana* transgenic lines by “floral dip” method (Zhang et al., 2006). Transformed plants were selected on MS 0.5X medium supplemented with 50 μg/mL hygromycin B. For this study, T4 generation homozygous plants were analyzed.

### RNA isolation and qRT-PCR analysis

The RNA isolation from Ws, amiR:*ADC*-L1, and -L2 15-day-old seedlings was carried out using Concert reagent (Invitrogen) according to manufacturer's instructions. The genomic DNA was removed through DNAse Turbo (Ambion, Austin, TX, USA) treatment. Finally, RNA concentration was quantified using an Epoch 2 microplate reader (Biotek Instruments, Winnoski, USA).

The synthesis and quantification of cDNA were performed through iScriptTM kit, One-Step RT-PCR kit, and the SYBR Green fluorophore, respectively (Applied Biosystems, USA). The qRT-PCR was performed in a 10 μL reaction mixture containing 50 ng of total RNA as template, 5 μL Power SYBR Green RT-Mix (2X), 200 nM oligonucleotides, and 0.08 μL RT enzyme mix (125X) using the direction system StepOne Real-Time PCR with the StepOne V2.1 software (Applied Biosystems, USA) as described previously (Rodríguez-Hernández et al., 2014; Ortega-Amaro et al., 2015). The gene expression was analyzed with the following conditions: 30 min at 48°C (cDNA synthesis), 10 min at 95°C (activation of AmpliTaq Gold DNA) following by 40 cycles of PCR for 15 s at 95°C (denaturation) and 1 min at 60°C (annealing/extension). Melting curves were performed with cycles of 15 s at 95°C (denaturation), 15 s at 60°C (alignment), and 15 s to 95°C (denaturation), incrementing 0.3°C per cycle. The *A. thaliana* ubiquitin 5 (*UBQ5*) gene was used as expression control. The relative gene expression levels of At*ADC1* and At*ADC2* genes were presented as 2^−ΔCt^, where ΔCt = Ct_*AtADC1* or *AtADC2*_-Ct_*UBQ5*_ (Livak and Schmittgen, 2001). The oligonucleotides that we used are described in Supplemental Table 1. For each sample, three biological replicates (*n* = 3) were analyzed with their respective technical replicates. Experiments were repeated at least twice with similar results.

### Free polyamines extraction and quantification

The *A. thaliana* free polyamines (Put, Spd, and Spm + tSpm) were determinated as dansyl-derivates by reversed phase HPLC (high performance liquid chromatography) approach. PAs extraction was done with 0.2 g plant material homogenized in 0.8 mL 5% (v/v) perchloric acid and incubated overnight at 4°C. After centrifugation, 0.2 mL of the supernatants was dansylated in a mixture containing 0.1 mL of saturated Na_2_CO_3_, 0.2 mL dansylchloride (5 mg/mL acetone), and 1 mM 1,7-diaminoheptane as internal standard. The mixture was incubated overnight in darkness at room temperature. Reaction was stopped by adding 0.1 mL proline (100 mg/mL), and dansylated PAs were extracted with 0.4 mL toluene. Finally, the organic phase was vacuum-evaporated and dansylated PAs were dissolved in 0.05 mL acetonitrile.

Plant PAs were analyzed by HPLC using a 4.6 × 150 mm C18 reverse phase column, in which the flow was 1.5 mL/min, and the elution gradient was prepared with eluent A (water) and eluent B (acetonitrile). Then, it was equilibrated with 70% B and 30% A before injecting 0.01 mL samples. This was followed by a linear gradient ending with 100% B during 9 min. The final step was held for 4 min before regenerating the column. Detection was done with a fluorometer using excitation and emission wavelengths of 415 and 510 nm, respectively, according to Flores and Galston (1982). A relative calibration procedure was used to determine the PAs in the samples, using 1,7-diaminoheptane as the internal standard and PA concentrations ranging from 0.3 to 1.5 nmol (Sigma-Aldrich Mexico). The results were expressed as nmoles per gram of fresh weight (nmol/gFW).

### Measurement of fresh weight, primary root length, lateral root number, and seed weight

The *A. thaliana* seeds from Ws, *adc1, adc2*, and amiR:*ADC*-L2 lines were grown on 0.5X MS plates for 7 and 14 days. Fresh weight (mg) of seedlings was obtained on an analytical scale and the values obtained represent the means from seven groups comprised of four seedlings per line (*n* = 7). Subsequently, the estimation of primary root length (cm) was determined by measuring the length of 28 roots of each line (*n* = 28). The image analysis software IMAGE J (http://rsb.info.nih.gov/ij) was used to evaluate primary root length. The lateral root number was measured using a stereomicroscope Motic SMZ-143 (*n* = 28). Seed weight was calculated from three replicates for each line (Ws, *adc1, adc2*, and amiR:*ADC*-L2), where 200 seeds represent each replicate (*n* = 3). In particular for amiR:*ADC*-L2 line seeds, 200 yellow and 200 brownish seeds were estimated. Each seed lot (time post-harvest) was measured on an analytical scale, and weights are expressed in mg. All measurements were repeated at least twice times with similar results.

### Measurement of flowering time

Ten days-old Ws, *adc1, adc2*, and amiR:*ADC*-L2 seedlings grown on 0.5X MS plates were transferred to soil pots into a growth chamber under 16 h of 120 μmol m-^2^ s-^1^ light and 8 h of darkness. The flowering time was estimated by recording the number of days after sowing in which the inflorescence reached 1 cm in length. The flowering time data was represented as the percentage of plants with floral tissues (*n* = 10).

### Exogenous polyamine application

The Ws and amiR:*ADC*-L2 stratified seeds (2 days at 4°C), were germinated and grown on 0.5X MS plates into a growth chamber for 7 days. After, the seedlings were transferred to divided plates, containing both 0.5X MS medium and 0.5X MS supplemented with 10 μM each PA (Put, Spd, and Spm). The plates were incubated into a grown chamber for 9 days. Subsequently, the mean of fresh weight and primary root length per plant were calculated (*n* = 15). In the case of flowering rate recovering, amiR:*ADC*-L2 14-day-old seedling grown on 0.5X MS plates were transferred to soil pots and their aerial tissues were sprayed with a solution containing 10 μM Put for 15 days.

### Microscopic analyses

The immature and mature siliques from Ws, *adc1, adc2*, and amiR:*ADC*-L2 were dissected from the plant, and the valves were gently removed from siliques using forceps and scalp. The septum and seeds were observed under a stereomicroscope (Motic SMZ-143). The magnification for the images captured was 15x to mature siliques, and 40x for immature siliques. Ten immature and mature siliques of each line were analyzed, and a representative image is shown in each column. For scanning electron microscopy (SEM) analysis, dry seeds were glued onto pure carbon containing polymer films and they were examined using a high-resolution scanning electron microscope (FEI ESEM-QUANTA 200; Low Vacuum/Water) operating at 25 kV and 90–100 Pa. Micrographs were digitalized using the basic photo program from the OS X system (Apple, CA, USA). Ten seed and embryos of each line were analyzed, and a representative image is shown in each column.

### Statistical analyses

One-Way Analysis of Variance (ANOVA) and Tukey's post-test analyses were performed to assess statistical significance. The GraphPad Prism version 5.0b (GraphPad Software, San Diego, California, USA) was used. Data represent the mean ± SEM. Differences at *P* < 0.05 were considered significant.

## Results

### Silencing of *A. thaliana ADC1* and *ADC2* genes by artificial microRNA

**Figure 1:**
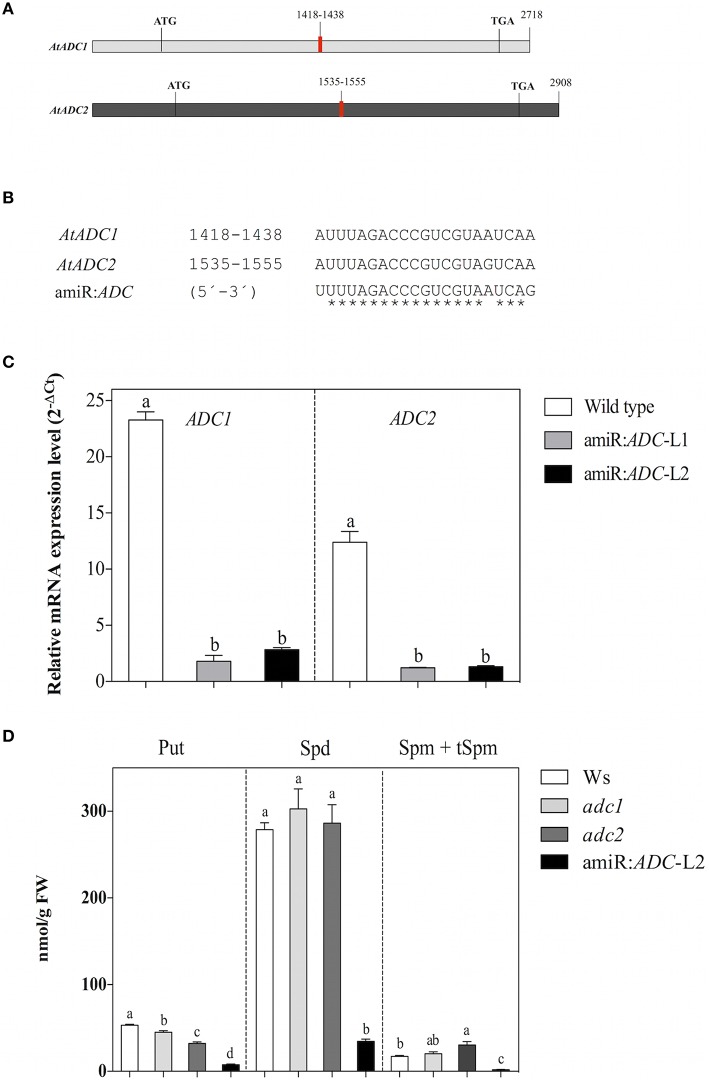
**The artificial microRNA amirR:***ADC*** is effective to silence both ***ADC*** genes and decreased the polyamine levels of ***A. thaliana*****. **(A)** Schematic representation of *AtADC1* and *AtADC2* transcripts. Target regions of the artificial microRNA are indicated in red. **(B)** Multiple alignments of *AtADC1* and *AtADC2* target region with the predicted mature amiR:*ADC* sequence (5′-3′). **(C)** Transcript levels of *AtADC1* and *AtADC2* genes estimated in Ws, amiR:*ADC*-L1, and -L2 lines by qRT-PCR approach. The *A. thaliana ubiquitin 5* (*UBQ5*) gene was used as expression control and the relative gene expression levels of *AtADC1* and *AtADC2* genes were presented as 2^−ΔCt^, where ΔCt = Ct_*AtADC*1_ or Ct_*AtADC2*_-Ct_*UBQ*5_. **(D)** Quantification of free polyamine levels [putrescine (Put), spermidine (Spd), and spermine + thermospermine (Spm + tSpm)] in Ws, *adc1, adc2*, and amiR:*ADC*-L2 12-day-old seedlings using HPLC technique. Error bars denote SE (*n* = 3) and different letters are used to indicate means that differ significantly according to the One-way ANOVA analysis and Tukey's multiple comparison test (*P* < 0.05).

In order to simultaneously silence both *ADC* genes from the *A. thaliana*, we generated an artificial microRNA (amiR:*ADC*) using the Web MicroRNA Designer (WMD; Schwab et al., 2006; Ossowski et al., 2008). The designed amiR:*ADC* is targeted to consensus region of 21 nucleotides between the *AtADC* genes (*AtADC1*:1418–1438 bp and *AtADC2*: 1535–1555 bp; Figure 1A). As is shown in the alignment, exist one mismatch at position 17 for *AtADC2* target, and other mismatch for both mRNAs at 1 and 21 positions (Figure 1B). These mismatches should reduce potential transitive effects due to priming and extension by RNA dependent RNA polymerase (Ossowski et al., 2008). Besides, there is no mismatch at the cleavage site (position 10–11). We analyzed the expression levels of each *AtADC1* and *AtADC2* genes by qRT-PCR approach in the generated *A. thaliana* transgenic lines. In this sense, a strong reduction of both *AtADC* transcript levels was observed in the amiR:*ADC*-L1 and -L2 transgenic lines (8 to 9.6 fold for *AtADC1* and 6 fold for *AtADC*2) compared to the expression of wild type (Ws) seedlings (Figure 1C). Our data show that the expression of designed amiR:*ADC* reduce the transcript levels from both *A. thaliana AtADC1* and *AtADC2* genes. For the following experiments amiR:*ADC*-L2 line was used.

### The amiR:*ADC* seedlings show reduced polyamine levels

Since *AtADC1* and *AtADC2* are rate-limiting enzymes for PA biosynthesis, we explored whether the silencing led to a decrease in PA levels. We quantified the total free PAs content (Put, Spd, and Spm + tSpm) in the Ws, *adc1* and *adc2* single mutants, and amiR:*ADC*-L2 12-day-old seedlings using HPLC technique. Our analyses reveal that the amiR:*ADC*-L2 line contain the lowest PA levels in contrast to all lines evaluated; even, the Put levels were lower than in the *adc2* mutant, which it has been known to show a reduction of their Put levels (Urano et al., 2004). In particular, the Put, Spd, and Spm + tSpm levels were reduced 85, 88, and 89%, respectively, compared to the parental Ws (Figure 1D). These results indicate that the simultaneous silencing of *A. thaliana AtADC* genes impact severely in their PAs content.

### The amiR:*ADC* seedlings display a stunted growth phenotype

**Figure 2:**
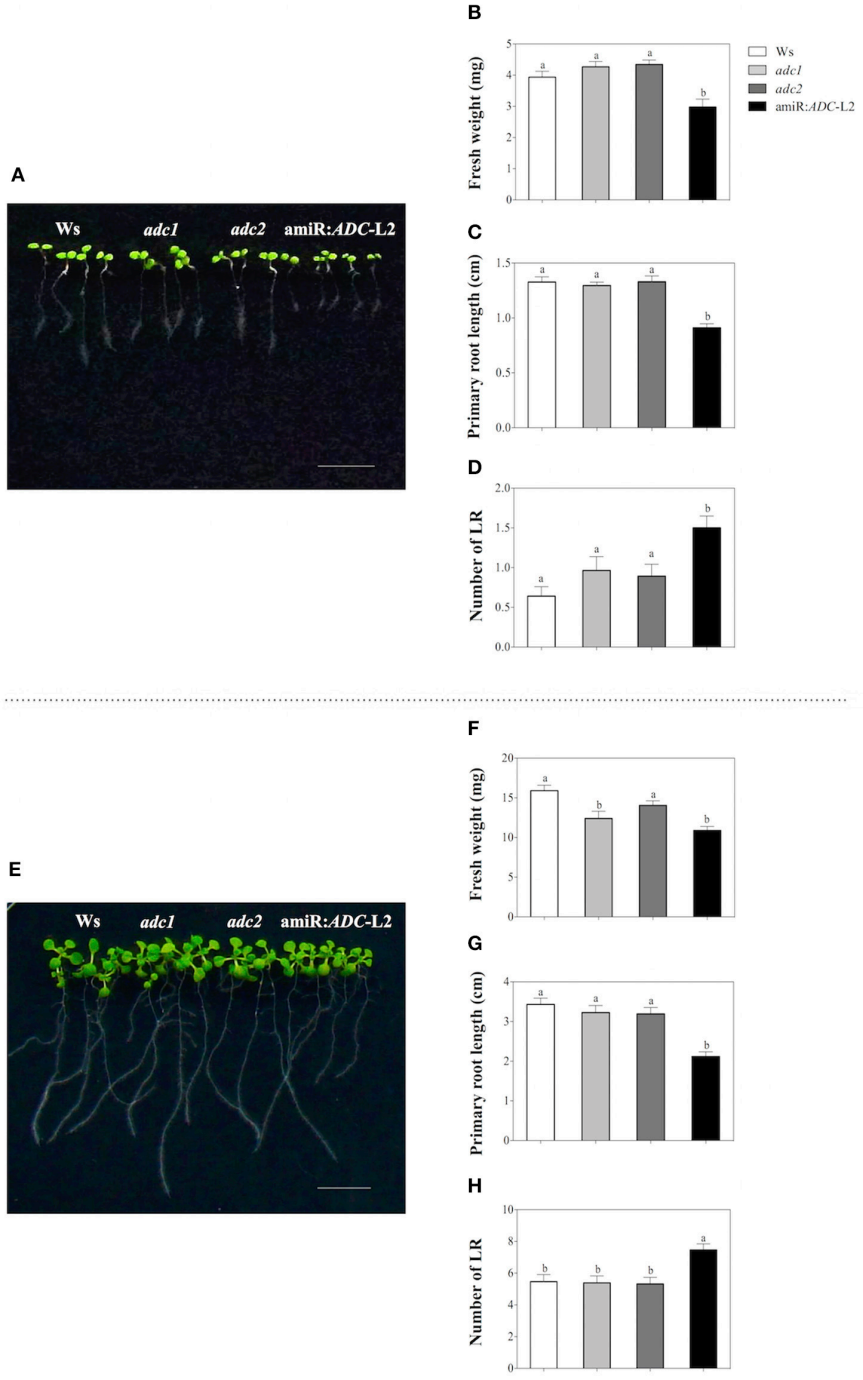
**Stunted growth of the amiR:***ADC***-L2 seedlings. (A,E)** Phenotype of 7-and 14-day-old Ws, *adc1, adc2*, and amiR:*ADC*-L2 seedlings grown on MS plates, respectively. The scale bar corresponds to 1 cm. Measure of fresh weight **(B,F),** primary root length **(C,G)**, and number of lateral roots (LR) **(D,H)**. To fresh weight the values represent means of seven groups of four seedlings (*n* = 7). To primary root length and number of lateral roots (LR) error bars represent means SE (*n* = 28) and different letters are used to indicate means that differ significantly according to the One-way ANOVA analysis and Tukey's multiple comparison test (*P* < 0.05).

In order to evaluate the physiological roles of diminished PA level in amiR:*ADC*-L2 line, we have analyzed the growth rate, such as fresh weight, primary root length, and lateral root number of the Ws, *adc1, adc2*, and amiR:*ADC*-L2 seedlings grown on MS medium. Seven days-old amiR:*ADC*-L2 seedlings showed 25% fresh weight reduction in contrast to the wild type Ws (2.9 ± 0.2 and 3.9 ± 0.2 mg, respectively; Figures 2A,B); however, significant differences were not found in the fresh weight of *adc1* and *adc2* lines respect to Ws (Figure 2B). Likewise, the amiR:*ADC*-L2 seedlings disclosed impaired development, which is reflected in a 30% reduction of primary root length compared to parental Ws (0.9 ± 0.03 and 1.3 ± 0.04 cm, respectively). In contrast, there was no difference in the main root length of *adc1* and *adc2* lines (Figure 2C). Additionally, we observed a significant augment of the lateral roots number in amiR:*ADC*-L2 (1.5 ± 0.2) in comparison to Ws (0.6 ± 0.1), the *adc1, adc2* lines (1 ± 0.2 and 0.9 ± 0.1, respectively) showed increment of lateral root number compared to the parental line (Figure 2D).

When the plantlets were 14 days old, we observed similar behavior in the physiological parameters that were measured (Figure 2E). The amiR:*ADC*-L2 plantlets reduced their fresh weight in 31% respect to the Ws plantlets (11 ± 0.5 and 16 ± 0.7 mg, respectively). Also, a significant difference was found in the fresh weight of *adc1* (12 ± 0.9 mg) in comparison to Ws (Figure 2F). The primary root length was diminished 38% in the amiR:*ADC*-L2 line compared to the Ws (2.1 ± 0.1 and 3.4 ± 0.1, respectively; Figure 2G). Noticeably, the lateral roots number was higher in the amiR:*ADC*-L2 than Ws, *adc1*, and *adc2* lines (Figure 2H). Additionally, we evaluated the independent transgenic line amiR:*ADC*-L1, which showed a slightly reduction of the primary root length in 7 days and it increased the number of lateral roots in 7 and 14 days. However, this line did not present the pronounced phenotype that was observed in the amiR:*ADC*-L2 line (Supplemental Figure S1). These data suggest that the PA homeostasis is necessary for normal early development in *A. thaliana*.

### PAs addition rescued the stunted growth of amiR:*ADC* seedlings

**Figure 3:**
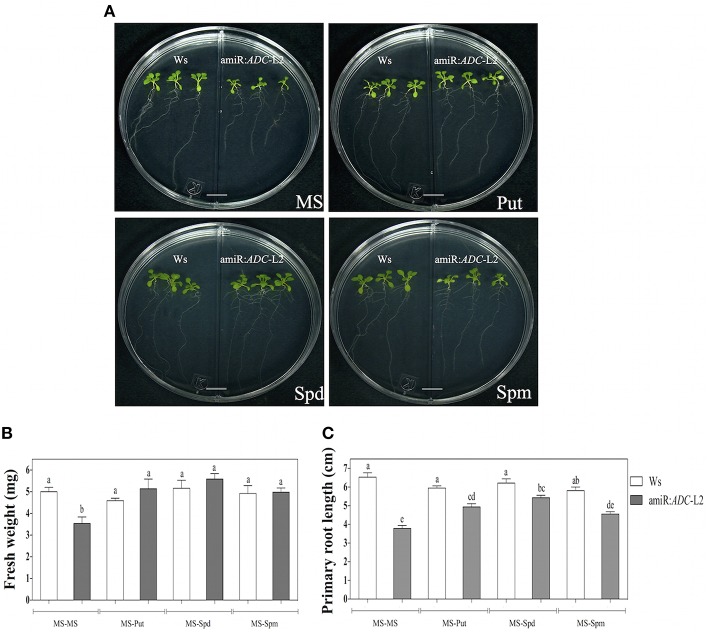
**Polyamine application alleviates the stunted phenotype of amiR:***ADC***-L2 seedlings**. **(A)** Ws, *adc1, adc2*, and amiR:*ADC*-L2 16-day-old seedlings were grown on split plates containing both MS medium (left side) and MS supplemented with 10 μM Put, Spd, or Spm (right side). The scale bar corresponds to 1 cm. Measure of fresh weight **(B)**, and primary root length **(C)** of the plantlets. The mean of fresh weight is presented as mg per plant. Error bars denote SE (*n* = 15) and different letters are used to indicate means that differ significantly according to the One-way ANOVA analysis and Tukey's multiple comparison test (*P* < 0.05).

In addition, we were interested in exploring if the stunted growth was reverted by the exogenous addition of PAs. For this purpose, amiR:*ADC*-L2 7-day-old seedlings were grown in the right side of split Petri dishes in presence of 10 μM Put, Spd, or Spm (Figure 3A). In the left side of the split dish, the Ws seedlings without PA treatment were grown as control. Both physiological parameters: fresh weight and primary root length (Figures 3B,C, respectively) increased in the silenced plants after Put, Spd or Spm treatments, being able to reach the parental levels. However, discrete differences were observed between PAs, as the Spm generated the lower recovery in the primary root length in comparison to Put and Spd treatments.

### Co-silencing of *ADC* genes alters flowering rate in *A. thaliana*

**Figure 4:**
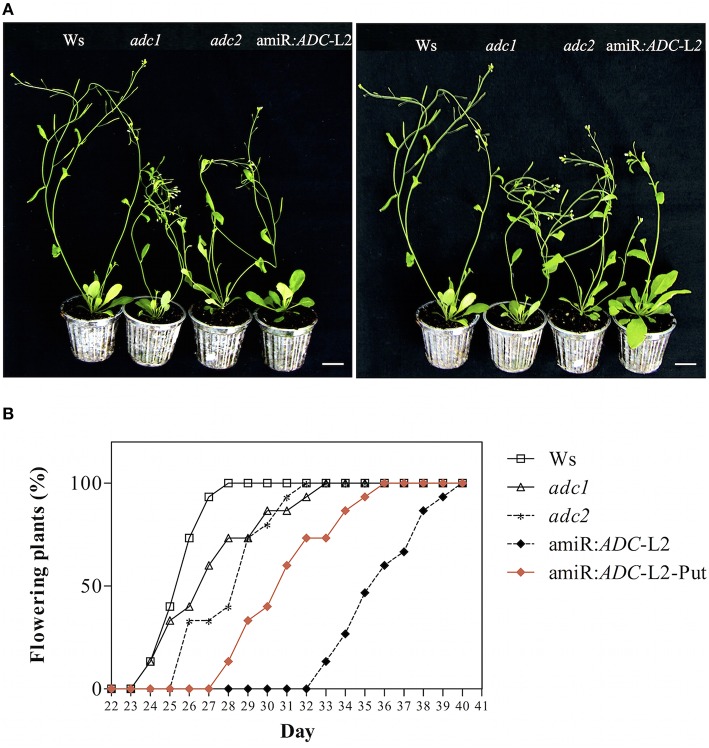
**The amiR:***ADC***-L2 plants are altered in their flowering rate. (A)** Phenotype of 35-day-old Ws, *adc1, adc2*, and amiR:*ADC*-L2 plants with or without putrescine (Put) treatment grown on soil pots under long day conditions. The plants were sprinkled with 10 μM Put for 15 days. The scale bar corresponds to 2.5 cm. **(B)** Percentage of flowering rate of Ws, *adc1, adc2*, and amiR:*ADC*-L2 plants under long day conditions (*n* = 10). The percentage of flowering rate recovering is showed with red rhombus in **(B)**.

We were able to find a visible change from flower initiation in the amiR:*ADC*-L2 transgenic line. We found that silenced plants showed a delay in flowering time (up to 9 days) relative to Ws under long day conditions (Figures 4A,B). In addition, the *adc2* mutant line displayed a slight delay of 2 days in the inflorescence emergence in comparison to Ws (Figure 4B). Previously, we observed that the stunted growth in 16-day-old amiR:*ADC*-L2 seedlings was reverted with the applying of Put (Figure 3). Consequently, we analyzed whether the flowering time could be recovered after Put treatment (Figure 4). In this sense, 14-day-old amiR:*ADC*-L2 seedlings that were sprinkled with 10 μM Put for 15 days showed an reduce of 5 days in their flowering time in comparison to non-treated plants (Figures 4A,B).

**Figure 5:**
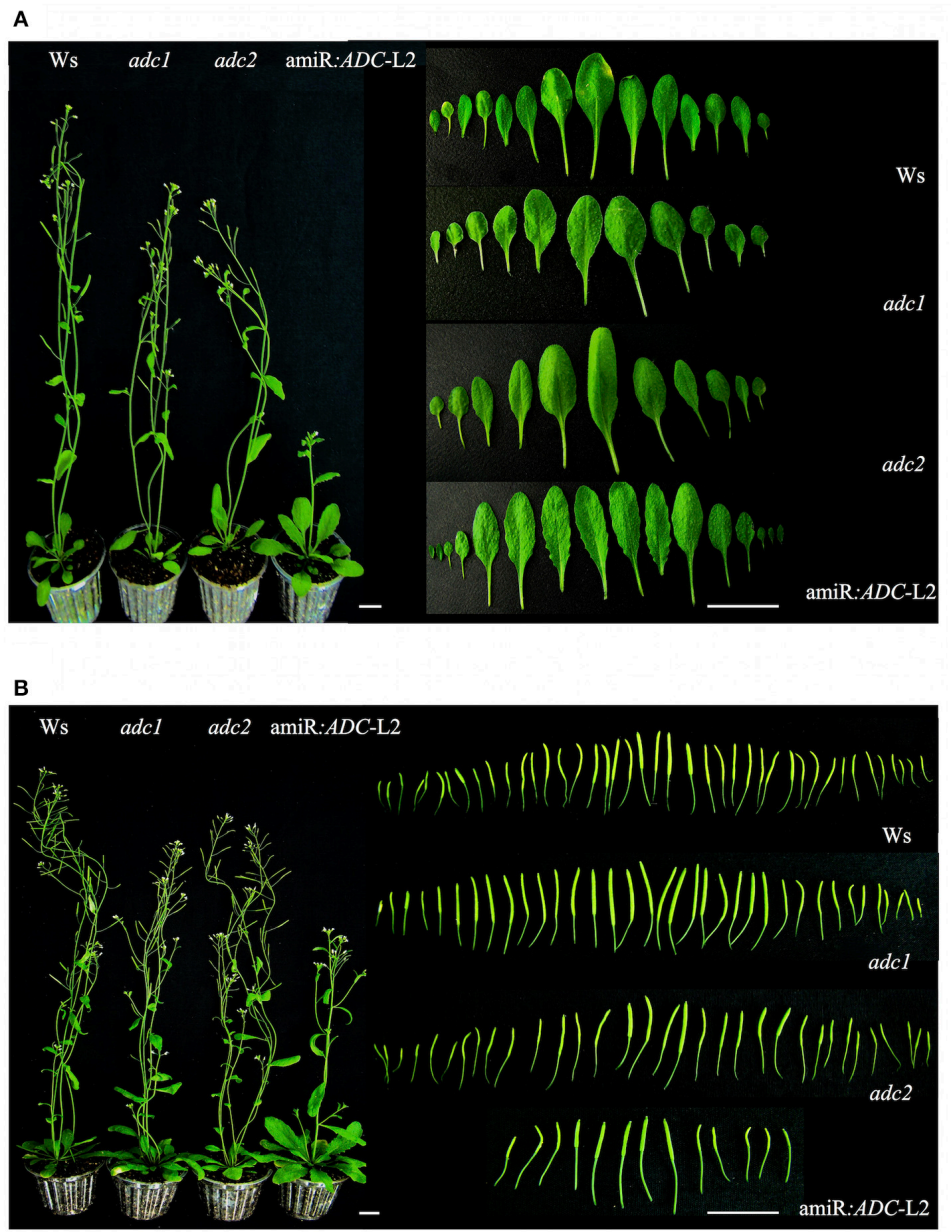
**Morphology analysis of amiR:***ADC***-L2 45- and 55-day-old plants. (A)** Plant and rosette leaves phenotypes of 45-day-old Ws, *adc1, adc2*, and amiR:*ADC*-L2 plants grown under long day conditions. In the right panel the shape fully expanded of abaxial surface from rosette leaves are shown. **(B)** Plant phenotype and silique number of 55-day-old Ws, *adc1, adc2*, and amiR:*ADC*-L2 plants grown under long day conditions. The right panel shows the total number of immature silique at 55 day. The scale bars correspond to 2.5 and 1 cm (left and right panel, respectively).

Afterwards, we investigated the phenotypic characteristics of 45- and 55-day-old amiR:*ADC*-L2 plants growth under a long day condition (Figure 5). At 45 days, the amiR:*ADC*-L2 plants remained with the slow growth pattern (Figure 5A). Another feature observed in the amiR:*ADC*-L2 mutant line was the serrate rosette leaves (Figure 5A). Furthermore, we examined the number of siliques in 55 day-old-plants. Our data shows that the amiR:*ADC*-L2 had a low number of siliques at this stage compared to the wild type Ws (Figure 5B).

### The seed development is affected by co-silencing of *ADC* genes

**Figure 6:**
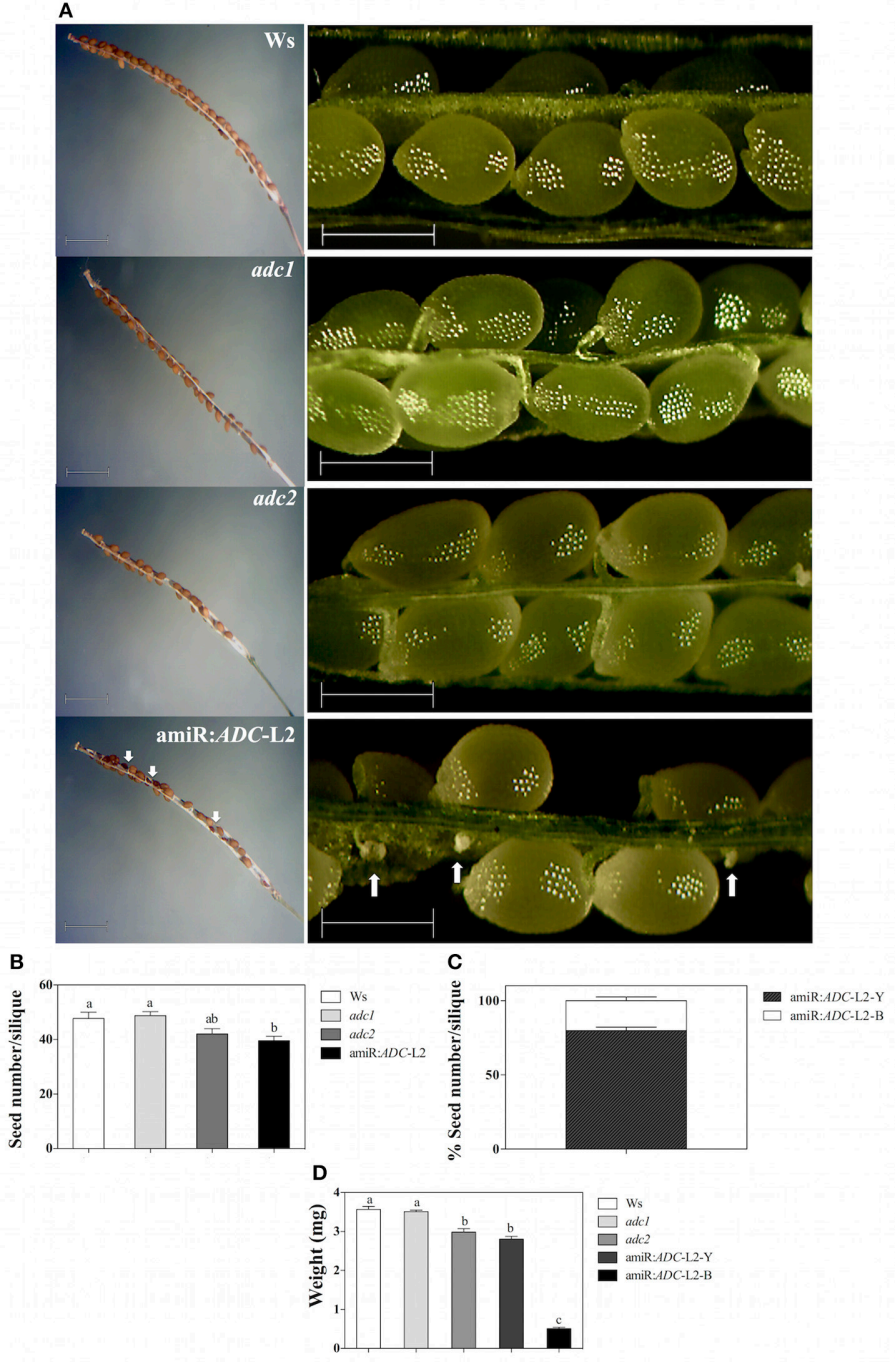
**The seed development is affected in amiR:***ADC***-L2 plants. (A)** Mature and immature siliques (left and right panel, respectively) from 55-day-old Ws, *adc1, adc2*, and amiR:*ADC*-L2 plants grown under long day conditions. The white arrows indicate the abortive seeds distribution in amiR:*ADC*-L2 siliques. In the left and right panels the scale bars correspond to 1500 and 500 μm, respectively. **(B)** Seed number per mature silique from 55-day-old plants. **(C)** Percentage of both yellow (Y) and brownish (B) seeds per silique in the amiR:*ADC*-L2. The measures were carried out using 10 siliques per plant of 10 plants (*n* = 10). **(D)** Weight of 200 seeds from mature siliques of Ws, *adc1, adc2*, and amiR:*ADC*-L2. To amiR:*ADC*-L2 lines and their normal (Y) and abortive (B) seeds were separated and analyzed individually (*n* = 3). Error bars denote SE and different letters are used to indicate significant differences among lines according to the One-way ANOVA analysis and Tukey's multiple comparison test (*P* < 0.05).

We found a striking phenotype in amiR:*ADC*-L2 seeds, suggesting that the seed development is also altered in the silenced plants. As we can notice in the Figure 6A, stereomicroscope analyses showed that amiR:*ADC*-L2 immature and mature siliques had some abortive seeds. Because it was clear that the seeds were altered in amiR:*ADC* siliques, we were interested in analyzing the number of seeds and weight. Our data showed approximately 20% reduction of seed number in amiR:*ADC*-L2 siliques compared to parental Ws (40 ± 2 and 48 ± 2 seed number per siliques, respectively; Figures 6A,B); similarly, the *adc2* single mutant produced less seeds per silique (42 ± 2; Figure 6B). Stereomicroscope analyses reveal that both amiR:*ADC*-L2 immature and mature siliques contain a 80% of normal seeds (large and yellow) and a 20% of abortive seeds (shrunken and brownish; Figures 6A,C). The phenotype of abortive seeds in mature siliques from amiR:*ADC*-L2 was reverted by exogenously addition of Put (Supplemental Figure S2). Additionally, we analyzed the weight of 200 seeds of each line, including the 2 types of seeds of the amiR:*ADC*-L2. The weight of Ws and *adc1* mutant seeds did not present significant differences between them. In contrast, the weight of *adc2* mutant and amiR:*ADC*-L2 yellow seeds had decreased 16 and 21%, respectively, compared to the Ws (Figure 6D). In addition, the reduction in the brownish seeds, which had severe defects in cell morphology, recorded a weight of 0.5 ± 0.03 mg per 200 seeds in comparison to 3.5 ± 0.07 mg in Ws (Figure 6D).

**Figure 7:**
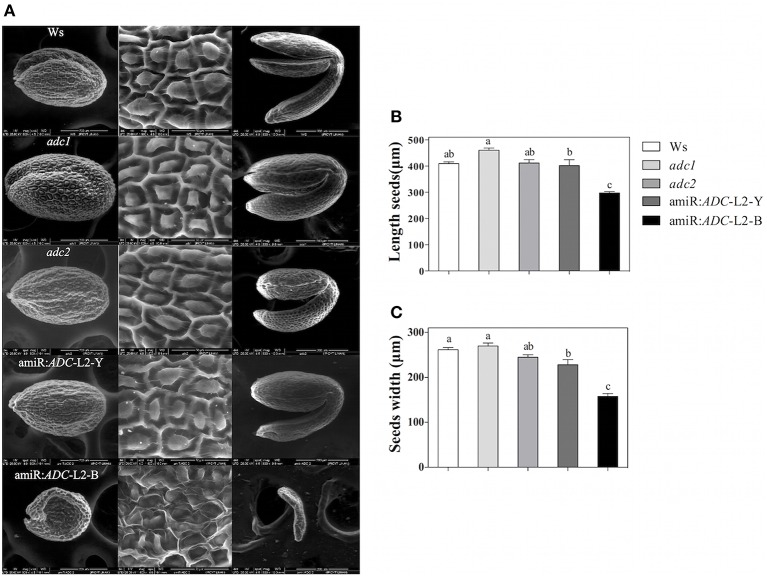
**Seed and embryo morphology alterations of amiR:***ADC***-L2**. **(A)** Environmental scanning electron micrograph of seeds and embryos from Ws, *adc1, adc2*, and amiR:*ADC*-L2. The left and central panels show the coat of mature seed, and the right panel discloses their respective mature embryos. Ten seeds of each line were analyzed and representative images are shown in the columns. The scale bars correspond to 200 μm (left and right panels) and 50 μm (central panel). **(B)** Measure of length and **(C)** width of mature seeds. To amiR:*ADC*-L2 their normal (Y) and abortive (B) seeds were separated and analyzed individually. Error bars denote SE (*n* = 10) and different letters are used to indicate significant differences among length and width seed according to the One-way ANOVA analysis and Tukey's multiple comparison test (*P* < 0.05).

Finally, the scanning electron microscopy (SEM) approach was used to analyze the external morphology alterations in amiR:*ADC*-L2 seeds. The micrographs revealed that the cells surface from Ws, *adc1, adc2*, and amiR:*ADC*-L2 (yellow) seeds showed similar polygonal structures and central elevation, in contrast to brownish seeds of amiR:*ADC*-L2 that showed an irregular shape (Figure 7A). In particular, the brownish seeds exhibited a reduction of 28 and 40% in length and width seed, respectively, compared to Ws (Figures 7B,C). We therefore examined the embryo development of amiR:*ADC*-L2 seeds and found that the brown seeds had a late arrest in development of embryos corresponding at torpedo stage (Figure 7A).

## Discussion

Plant growth and development processes involve environmental and endogenous signals that contribute to determine the anatomy, physiology and molecular features of the plant. Within the endogenous signals, the polyamines (PAs) have been recognized in higher plants as key players in these biological processes (Mattoo et al., 2010; Takahashi and Kakehi, 2010; Tiburcio et al., 2014). Moreover, the literature indicates that PAs are implicated in a variety of cellular processes, including control cell division, root formation, flowering, retardation of senescence, and response to stress (Watson et al., 1998; Pandey et al., 2000; Alcázar et al., 2005; Jiménez-Bremont et al., 2007; Ortega-Amaro et al., 2012).

We generated a non-lethal *A. thaliana* line with very low levels of major polyamines by using an artificial microRNA amiR:*ADC*. An effective tool in plants to silence genes in a simultaneously manner, when the single or multiple mutants have a lethal phenotype, is made through an artificial microRNA because it provides the opportunity to analyze the mutant lines during life span. In this sense, the artificial miRNA design targets a consensus region in both *ADC* genes, and the fact that the two *ADC* genes in *A. thaliana* are attractive targets of genetic manipulation is because this plant lacks of the *ODC* gene (Hanfrey et al., 2001), thus biosynthesis *de novo* of PA is only through the ADC route. Plants with lowest levels of PAs are desired in order to provide further evidence of the importance of PA homeostasis in plant growth and development processes.

Important biochemical and genetic efforts have been performed in order to manipulate the *ADC* expression or activity that have negative impact in PAs levels and their association with defects in development. For example, in the latest 80's the DL-alpha difluoromethylarginine (DMFA), an irreversible inhibitor of ADC activity, was commonly used in plants (Slocum and Galston, 1985). Although the use of inhibitors has contributed to determine the role of PAs, they are often criticized due to their possible side effects. Furthermore, it was reported that the *A. thaliana* T-DNA insertion line for *AtADC1* gene did not show differences in their morphology and PA levels at 21-day-old in contrast to wild type plants (Urano et al., 2004). On the other hand, the T-DNA insertion line for *AtADC2* gene displayed significantly reduction of Put levels. However, their Spd and Spm levels were not reduced. Additionally, it was observed an increased sensibility to salt stress of *adc2* 21-day-old mutant line (Urano et al., 2005). Unfortunately, the double-knockout mutant of *AtADC1* and *AtADC2* genes was lethal at the embryo stage (Urano et al., 2005).

Regarding to this, the generated *A. thaliana* amiR:*ADC*-L2 transgenic line exhibited a stunted growth during its life span, which was mainly reflected in diminished of fresh weight seedling, primary root length, higher number of lateral roots, and delayed flowering time. Interestingly, amiR:*ADC*-L2 transgenic plantlets had a higher number of lateral roots in comparison to parental plantlets. In addition, we observed marginal serrations in rosette leaves and abortive seeds in amiR:*ADC* line. These data suggest that the silencing of *AtADC*1 and *AtADC*2 genes may impact in Arabidopsis physiological features. The major evidences in our work that suggest that these phenotypic hallmarks are related with the decrease in PA levels are (1) the data of PA profile obtained by HPLC in 12-day-old seedlings that is significant lower (more than 80%) for the three main PAs, and (2) the recovering growth parameters, such as fresh weight and root length of 16-day-old silenced seedlings, when PAs were applied exogenously. In this sense, Hanzawa (2000) demonstrated that the exogenous application of thermospermine can alleviate the stunned and strong dwarf phenotypes observed in *A. thaliana acl5* mutants.

Consistently, the delay flowering time in *A. thaliana* amiR:*ADC* transgenic line was also reverted when Put was applied exogenously. It has been reported that high Spd levels are related to floral initiation in *Nicotiana tabacum*, demonstrating that Spd levels are higher in flowers than in vegetative buds; moreover, the inhibition of Spd synthesis prevents floral initiation and promotes the formation of vegetative buds in tobacco (Kaur-Sawhney et al., 1988). Furthermore, the PA profile (Put, Spd, Spm, and tSpm) in different *A. thaliana* tissues, showed that the contents of the four PAs, were higher in flower organs than in mature leaves (Naka et al., 2010). Also, Urano et al. (2003) reported that the contents of Put, Spd, and Spm are higher in siliques, buds, and flowers.

Besides, we detected an altered morphology of the seeds reflected by lower weight and fewer seed number per silique in both *adc2* and amiR:*ADC A. thaliana* plants. In particular, the amiR:*ADC* siliques contain 20% of abortive seeds, in which the embryo was arrested in torpedo stage. As expected, this phenotype was reverted by exogenously addition of Put. In this sense, Urano et al. (2005) reported that the *A. thaliana* siliques from heterozygous double mutant of *ADC* genes contain 25% of abortive seeds; moreover, all the homozygous *ADC* muted seeds are unable to germinate since their embryos are arrested at the heart to torpedo transition stage. Also, they described the tissue-specific expression of the promoter of *AtADC1* and *AtADC2* genes and they found that *AtADC2* promoter was detected in siliques and through the early developmental stages of embryo, endosperm, and still active during embryo maturation; remarkably, the *AtADC1* promoter was not detected in these tissues. So it seems that *AtADC2* is more functional during embryo development in contrast to *AtADC1*. Nevertheless, the amiR:*ADC*-L2 line showed a more drastic negative phenotype in the seeds in comparison with the single mutant *adc2*. On the other hand, it has recently been reported in wheat that exogenous application of Spd and Spm increased the grain filling rate and weight (Liu et al., 2013). The importance of PAs during embryo maturation is also recognized by the facts that mainly accumulate during early stages of seed development (Urano et al., 2005).

In addition to double mutant *adc1/adc2*, the double *spds1*/*spds2* mutant is also lethal at embryo stages (Imai et al., 2004). Thus, it is clear that is not possible to generate a polyamine-auxotrophic plant with traditional approaches, so it seems that the *ADC* silencing via an artificial miRNA strategy can provide us with valuable information regarding to the minimum levels that are required for optimum plant development, and also for a more detailed description of the growth defects associated with this genetic background. In this study, the phenotypic characterization of the simultaneous silencing of two *ADC* genes in *A. thaliana* reinforces the role of PAs during growth and development. This genetic background will be a very useful tool for further molecular studies in order to enlighten and to give more information related to the specific functions of PAs during homeostasis in plant development processes, and in stress responses.

## Author contributions

DS, AC, AR, IM, and JJ designed and carried out the experiments, analyzed the results, and wrote the manuscript. KU and KS contributed scientific advice, and wrote the manuscript. All authors have read and approved the final manuscript.

### Conflict of interest statement

The authors declare that the research was conducted in the absence of any commercial or financial relationships that could be construed as a potential conflict of interest.
