# Supplementary material for: Simultaneous Silencing of Two Arginine Decarboxylase Genes Alters Development in Arabidopsis
Source: Front Plant Sci. 2016 Mar 14;7:300. doi: 10.3389/fpls.2016.00300 (PMC4789552; doi:10.3389/fpls.2016.00300)
Supplement: Supplementary file 1 [file Table1.DOCX]

| **Amplification** | **Oligonucleotide name** | **PCR product** | **Sequence (5´- 3´)** |
| --- | --- | --- | --- |
| amir:*ADC* construction | amiR:*ADC* (I) | amir:*ADC* | GATTTTAGACCCGTCGTAATCAGTCTCTCTTTTGTATTCC |
|  | amiR:*ADC* (II) |  | GACTGATTACGACGGGTCTAAAATCAAAGAGAATCAATGA |
|  | amiR:*ADC* (III) |  | GACTAATTACGACGGCTCTAAATTCACAGGTCGTGATATG |
|  | amiR:*ADC* (IV) |  | GAATTTAGAGCCGTCGTAATTAGTCTACATATATATTCCT |
| qRT-PCR | Fw-*Adc1* | *AtADC1* | CCTTGGCGTTTACTACTGCG |
|  | Rv-*Adc1* |  | CGGTGAAGATCAAAGACAGAGG |
|  | Fw-*Adc2* | *AtADC2* | GCCGTATCTTGCAACTGAGC |
|  | Rv-*Adc2* |  | TGCAACAACAAACCACACGA |
|  | Fw-*UBQ5* | *UBQ5* | TCGACGCTTCATCTCGTCCT |
|  | Rv-*UBQ5* |  | CGCTGAACCTTTCCAGATCC |

**Supplementary table 1. Oligonucleotides for amir:*ADC* construction and qRT-PCR analysis**
