# Supplementary figures and images for: Simultaneous Silencing of Two Arginine Decarboxylase Genes Alters Development in Arabidopsis
Source: Front Plant Sci. 2016 Mar 14;7:300. doi: 10.3389/fpls.2016.00300 (PMC4789552; doi:10.3389/fpls.2016.00300)

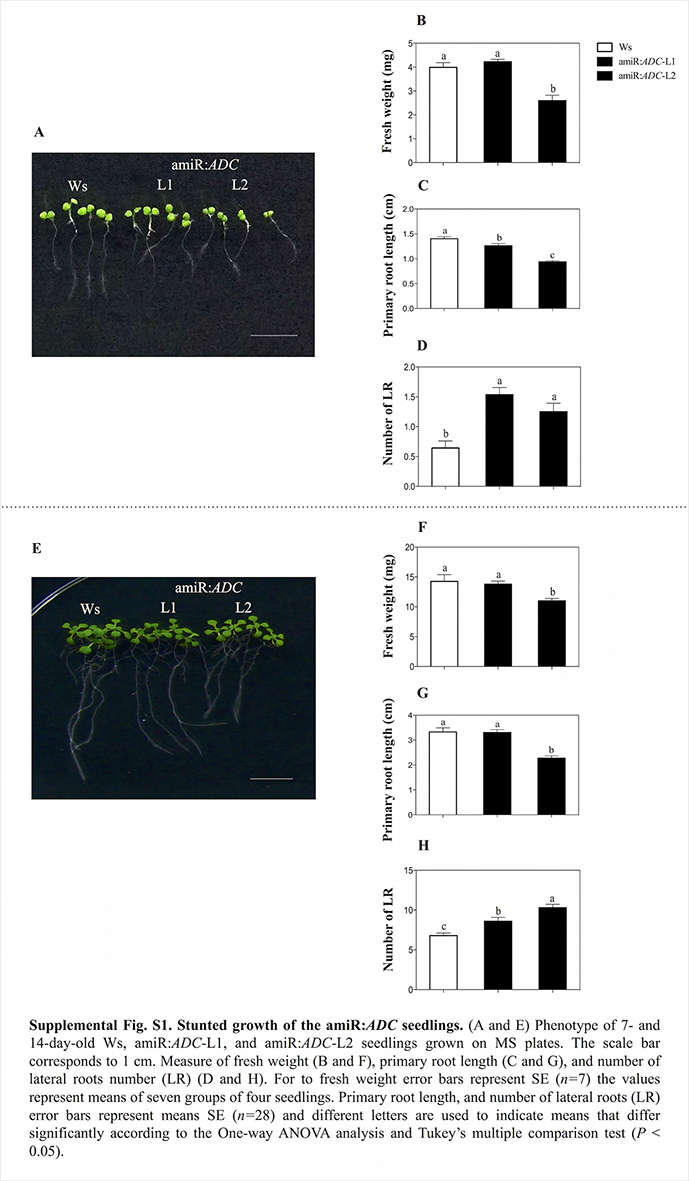

Supplement: Supplementary file 2 [file Image1.tiff]

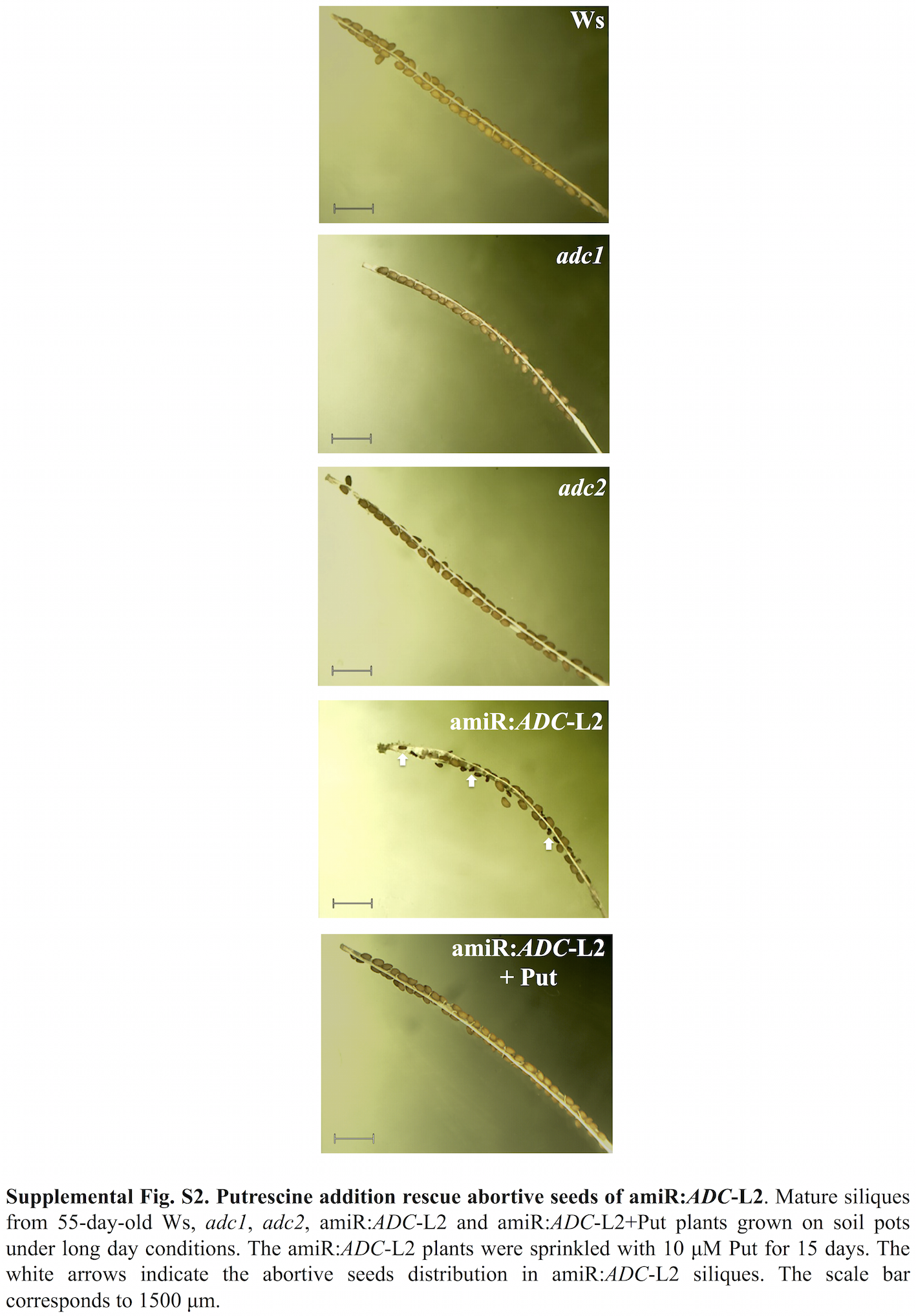

Supplement: Supplementary file 3 [file Image2.TIFF]
